# Ethyl pyruvate protects against sepsis-associated encephalopathy through inhibiting the NLRP3 inflammasome

**DOI:** 10.1186/s10020-020-00181-3

**Published:** 2020-06-09

**Authors:** Xiaoli Zhong, Lingli Xie, Xiaolong Yang, Fang Liang, Yanliang Yang, Jianbin Tong, Yanjun Zhong, Kai Zhao, Yiting Tang, Chuang Yuan

**Affiliations:** 1 Department of Hematology and Critical Care Medicine, The 3rd Xiangya Hospital, Central South University, Changsha, Hunan Province 410000 P. R. China; 2 Department of Pathophysiology, School of Basic Medical Science, Central South University, 138 Tong-zi-po Road, Changsha, Hunan Province 410000 P. R. China; 3 Department of Physiology, School of Basic Medical Science, Central South University, Changsha, Hunan Province 410000 P. R. China; 4 Department of Anesthesiology, Third Xiangya Hospital of Central South University, Changsha, Hunan China; 5 ICU Center, The Second Xiangya Hospital, Central South University, No. 139 Renmin Middle Road, Furong, Changsha, 410011 Hunan China

**Keywords:** Sepsis-associated encephalopathy, Ethyl pyruvate, NLRP3 inflammasome, Innate immunity, Sepsis

## Abstract

**Background:**

With the advance of antibiotics and life support therapy, the mortality of sepsis has been decreasing in recent years. However, the incidence of sepsis-associated encephalopathy (SAE), a common complication of sepsis, is still high. There are few effective therapies to treat clinical SAE. We previously found that ethyl pyruvate (EP), a metabolite derivative, is able to effectively inhibit the NLRP3 inflammasome activation. Administration of ethyl pyruvate protects mice against polymicrobial sepsis in cecal ligation and puncture (CLP) model. The aim of present study is to investigate if ethyl pyruvate is able to attenuate SAE.

**Methods:**

After CLP, C57BL/6 mice were intraperitoneally or intrathecally injected with saline or ethyl pyruvate using the sham-operated mice as control. New Object Recognition (NOR) and Morris Water Maze (MWM) were conducted to determine the cognitive function. Brain pathology was assessed via immunohistochemistry. To investigate the mechanisms by which ethyl pyruvate prevent SAE, the activation of NLRP3 in the hippocampus and the microglia were determined using western blotting, and cognitive function, microglia activation, and neurogenesis were assessed using WT, *Nlrp3*^*−/−*^ and *Asc*^*−/−*^ mice in the sublethal CLP model. In addition, *Nlrp3*^*−/−*^ and *Asc*^*−/−*^ mice treated with saline or ethyl pyruvate were subjected to CLP.

**Results:**

Ethyl pyruvate treatment significantly attenuated CLP-induced cognitive decline, microglia activation, and impaired neurogenesis. In addition, EP significantly decreased the NLRP3 level in the hippocampus of the CLP mice, and inhibited the cleavage of IL-1β induced by NLRP3 inflammsome in microglia. NLRP3 and ASC deficiency demonstrated similar protective effects against SAE. *Nlrp3*^*−/−*^ and *Asc*^*−/−*^ mice significantly improved cognitive function and brain pathology when compared with WT mice in the CLP models. Moreover, ethyl pyruvate did not have additional effects against SAE in *Nlrp3*^*−/−*^ and *Asc*^*−/−*^ mice.

**Conclusion:**

The results demonstrated that ethyl pyruvate confers protection against SAE through inhibiting the NLRP3 inflammasome.

## Introduction

Sepsis is a systemic inflammatory response syndrome (SIRS) caused by infection (Buras et al. 2005; Singer et al. 2016). Due to the advance of medicines and life-support techniques, the mortality of sepsis have been decreasing in recent years (Martin et al. 2003; Tanriover et al. 2006; Gaieski et al. 2013; Kaukonen et al. 2014; Stevenson et al. 2014; O’Neill 2016; Kaufmann et al. 2018). In addition to high mortality at the early stage, sepsis can cause sepsis-associated encephalopathy (SAE), which significantly increases the mortality of patients and largely influences the life quality of sepsis survivors (Eidelman et al. 1996; Ebersoldt et al. 2007; Widmann et al. 2014; Annane and Sharshar 2015). SAE is characterized by diffuse brain dysfunction after the onset of sepsis, without infection in the central nervous system (Eidelman et al. 1996; Ebersoldt et al. 2007; Chen et al. 2014; Widmann et al. 2014; Annane and Sharshar 2015). It has been reported that approximately 70% of survivors recovered from severe systemic infection have cognitive deficits (Iwashyna et al. 2010; Gofton and Young 2012). However, the underlying mechanisms of SAE are not fully uncovered and effective medicines for treating SAE are not available in clinics.

Previous studies have suggested that inflammatory responses, especially the inflammasome activation, is critical for the development of SAE (Yende et al. 2008; Erickson and Banks 2011; Annane and Sharshar 2015). Among different types of the inflammasomes, the NLRP3 (NLR family pyrin domain containing 3) inflammasome is the most well-characterized inflammasome (Hise et al. 2009) and contributes to the development of a number of monogenic autoinflammatory diseases, including the inherited CAPSs Muckle–Wells syndrome (MWS), familial cold autoinflammatory syndrome and neonatal-onset multisystem inflammatory disease (Masters et al. 2009), as well as various metabolic and neurodegenerative disorders(Wen et al. 2012), (De et al. 2014). However, whether pharmacological inhibition of the NLRP3 inflammasome could attenuate SAE is not known.

Pyruvate (CH3COCOO−), a key intermediate molecule in glucose metabolism, plays a protective role in many organ system damage models in vitro and in vivo (Bunton 1949; O'Donnell-Tormey et al. 1987; Salahudeen et al. 1991; Deboer et al. 1993; Nath et al. 1995; Cicalese et al. 1996; Crestanello et al. 1998; Dobsak et al. 1999). However, the poor stability in solution limits its therapeutic application in clinics (Montgomery and Webb 1956; Korff 1964). In 2001, Sims et al. discovered a stable derivative of metabolic intermediates pyruvate, named ethyl pyruvate (EP) (Sims et al. 2001), which is commonly used as a non-toxic food additive. Studies show that Ethyl pyruvate (EP) exerts protective effects in burn injury, shock, necrotizing pancreatitis and radiation-induced complications (Fink 2007) in a manner similar to pyruvate (Ulloa et al. 2002). In addition, EP treatment exerts an anti-inflammatory effect in various diseases such as sepsis (Miyaji et al. 2003; Sappington et al. 2003), alcoholic liver injury (Yang et al. 2003) and acute kidney injury (Salahudeen et al. 1991). We recently found that EP is a novel NLRP3 inhibitor (Li et al. 2018). However, whether EP treatment could improve cognitive function in SAE is still unknown. The aim of present study is to investigate the effect of EP in the SAE.

## Methods

### Animals

C57BL/6 (WT), *Nlrp3*^*−/−*^ and *Asc*^*−/−*^ male mice with age of 8–10 weeks and body weight of 20–25 g were used in the present study. C57BL/6 (H-2Kb, Thy-1.2) mice were purchased from Hunan SJA Laboratory Animal Co.Ltd. (Changsha, China). The *Nlrp3*^*−/−*^ mice and *Asc*^*−/−*^ mice (Mariathasan et al. 2004) were donated by Rongbin Zhou (CAS Key Laboratory of Innate Immunity and Chronic Disease, School of Life Sciences, University of Science and Technology of China). Mice were housed in the animal facility of Central South University and were maintained under standard condition (room temperature 22–25 °C with a 12-h light-dark cycle). Mice had free access to standard chow and water and had been acclimatized for at least 1 week before conducting experiments. Animal care and experimental procedures were performed with the approval from the Institutional Animal Care and Use Committees of Central South University.

### Sepsis model

#### Cecal ligation and puncture

After the mice anesthetized by 10 mg/kg xylazine hydrochloride and 200 mg/kg ketamine hydrochloride, a 1.5 cm longitudinal midline incision was made at the shaved and disinfected skin of lower quadrants of the abdomen and the cecum was exteriorized. The cecum was ligated at half between distal pole and the base of the cecum with 4–0 silk suture and a through-and-through puncture was made from mesenteric toward antimesenteric direction after medium ligation using 21-gauge needles. A small amount (droplet) of feces was extruded from both the mesenteric and antimesenteric penetration holes to ensure patency. The abdomen was closed and the mice were injected with pre-warmed normal saline (37 °C; 5 ml per 100 g body weight) subcutaneously to allow mice to recover from anaesthetization. Sham-operated animals were submitted to laparotomy and the cecum was taken out without puncture after laparotomy for sham operation.

### Intrathecal injections

Intrathecal injection was performed according to the protocol of Hayden and Wilcox (Hylden and Wilcox 1983). Anesthetized mice were slowly injected with 5 μL of PBS or EP between the L5 and L6 regions of the spinal cord using a 30-gauge needle 30 min after CLP operation.

### Behavioral tests

#### Open field test

As described previously, open field tests were carried out to evaluate the locomotor activity of mice (Zhang et al. 2013). To put it simply, the mice were gently placed in the center of the open field (50 × 50 cm). The movement of the mouse was recorded by computerized video tracking system (Logitech, Suzhou, China). The total traveled distance and average speed are analyzed by smart junior software 3.0 (Panlab, Cambridge, USA).

#### Novel object recognition

Novel object recognition experiment was carried out in a field arena of 20 cm × 30 cm × 30 cm. The test consists of two stages, namely, the training phase and the test phase (Bevins and Besheer 2006; Leger et al. 2013; Volmar et al. 2017; Briz et al. 2017). During the training phase, two identical objects are placed in symmetrical positions at equal distances from the center of the arena and from the walls of the arena. The mice were gently placed in the center of arena, with their heads opposite to the two identical objects, allowing them to explore freely for 10 min. Twenty-four hours post the training, one of the familiar items was replaced with a novel item, and the mouse was allowed to explore for 10 min in the arena. The objects and the chamber were cleaned with 75% alcohol solution between trials during training and testing. The preference index is defined as previous study (Qing et al. 2018).

#### Morris water maze test

The Morris water maze test was performed on the 15th day after the operation to evaluate spatial learning and memory of the mice. We used a computer video tracking system (Logitech, Suzhou, China) to record the movement of mice in water maze, according to previous research methods (He et al. 2012). To put it simply, a transparent circular platform is placed in the southwest quadrant of the circular pool 1 cm below the water surface. During the training period, the mice were first placed on the platform for 30 s to conform themselves to the environment, and then the mice were released into the water. In each experiment, mice were allowed to find the platform for a maximum of 60 s. If the mouse fails to find the platform within 60 s, it is guided to the platform and stays on the platform for 30 s. All mice were trained for 4 days, three times a day, changing releasing quadrant each trial, and the latency to platform was recorded in each experiment. After 4 days of training, take the platform out of the swimming pool on the fifth day. The mice were released into the water from the northeast quadrant. The movement trajectory of mice within 60 s was recorded. The memory ability of mice was evaluated by the number of platform crossings and the percentage of search time in the target quadrant.

### Immunostaining

The brains were obtained from the anesthetized mice. Half of the brain is used for immunostaining and the other half for Western blotting. The hemi-encephalon used for immunostaining was fixed overnight in 4% paraformaldehyde. After dehydration with sucrose, the brain was embedded in OCT and the coronal section (20 μm) of the brain was cut continuously by hypothermia thermostats. The slices were blocked with 5% BSA and 0.1% TritonX-100 for 1 h at room temperature, and incubated with rabbit polyclonal antibody (rabbit polyclonal antibody to ionized calcium binding adaptor molecule 1(IBA1):1:500, Wako, Japan, 10,904; rabbit polyclonal antibody to double cortin:1:500, CST, 4604) at 4 °C overnight. The slices were incubated in secondary antibody (1: 500) for 2 h. Three times of washes using 0.01 M PBS were conducted between each step. All the pictures were taken by microscope (Eclipse80i, Nikon, Japan) at the same light intensity and exposure time, and the executors were blind to the experimental conditions.

For immunofluorescence staining of ASC speck, mouse microglia loaded on 6-well slides were primed with ultra-pure LPS (100 ng/ml for 3 h. in the presence or the absence of EP (10 mM), and then stimulated with nigericin (10 μM) for 30 min. The microglia was fixed for 15mins in 4% paraformaldehyde, and blocked with 5% BSA and 0.1% TritonX-100 for 1 h at room temperature, followed by an incubation with rabbit polyclonal antibody (rabbit polyclonal anti-ASC antibody Adipogen AL177: 1:200, InvivoGen, A120-100D2) at 4 °C overnight. Afterward, the microglia were incubated in secondary antibody (1:500) for 2 h. Three times of washes using 0.01 M PBS were conducted between each step. All the pictures were taken by microscope (Eclipse80i, Nikon, Japan) at the same light intensity and exposure time, and the executors were blind to the experimental conditions.

### Western blot

To detect the NLRP3 and the DCX level of hippocampus (neonatal neurons), the frozen hippocampus was homogenized for proteins in a lytic buffer containing protease inhibitor cocktails (Roche, Germany, catalog number: 11873580001), followed by a centrifugation at 12,000 g for 20 min at 4 °C. The supernatant of the hippocampal homogenate was then collected. To determine the expression of NLRP3 and cleavage of pro-IL-1β in microglia, supernatant and cell lysates of microglia were analyzed using western blot. Cell-free supernatants were used to extracted proteins by methanol/chloroform precipitation as previously described (Wang et al. 2004; Li et al. 2018). Briefly, cell culture supernatants were precipitated by the addition of an equal volume of methanol and 25% volumes of chloroform, then were vortexed and centrifuged for 5 min at 20,000 g. The upper phase was discarded and 400 μl methanol was added to the interphase. After a centrifuge for 5 min at 20,000 g, the supernatants were removed. The protein pellet was dried at 55 °C for 2 min, and resuspended in Laemmli buffer and boiled for 10 min at 100 °C. Cell extracts were prepared as described previously (Wang et al. 2004). The protein samples were separated by SDS-PAGE and transferred to PVDF membrane (Millipore). After washing, 5% non-fat milk in TBST buffer was used to block the membrane for 1 h, and then incubated with primary antibodies (rabbit polyclonal antibody to double cortin: 1:1000, Cell Signaling Technology, catalog number: #4604; mouse anti-NLRP3 antibody:1:1000, AdipoGen, catalog number: AG-20B-oo14; goat anti-IL-1B polyclonal antibody:1:1000, RD systems, catalog number: AF-401-NA; Mouse anti-β-actin polyclonal antibody: 1:5000, Cell Signaling Technology, catalog number: #3700 s) overnight on 4°C. After washes for three times, the membranes were incubated with secondary antibody at room temperature for 2 h. Finally, the protein was visualized by Western Bright ECL-Spray (advansta, catalog number: K-12049-D50), and the intensity of each band was measured by density method.

### Statistical analysis

All statistical analyses were performed using GraphPad Prism Software (version6.0) and *P* < 0.05 was considered statistically significant. Analysis of Variance (ANOVA) and post-hoc test were used to compare more than two groups. The mean and Standard Error of Mean were calculated in experiments with multiple data points.

## Results

### Ethyl pyruvate attenuates cognitive decline in experimental sepsis

**Fig. 1:**
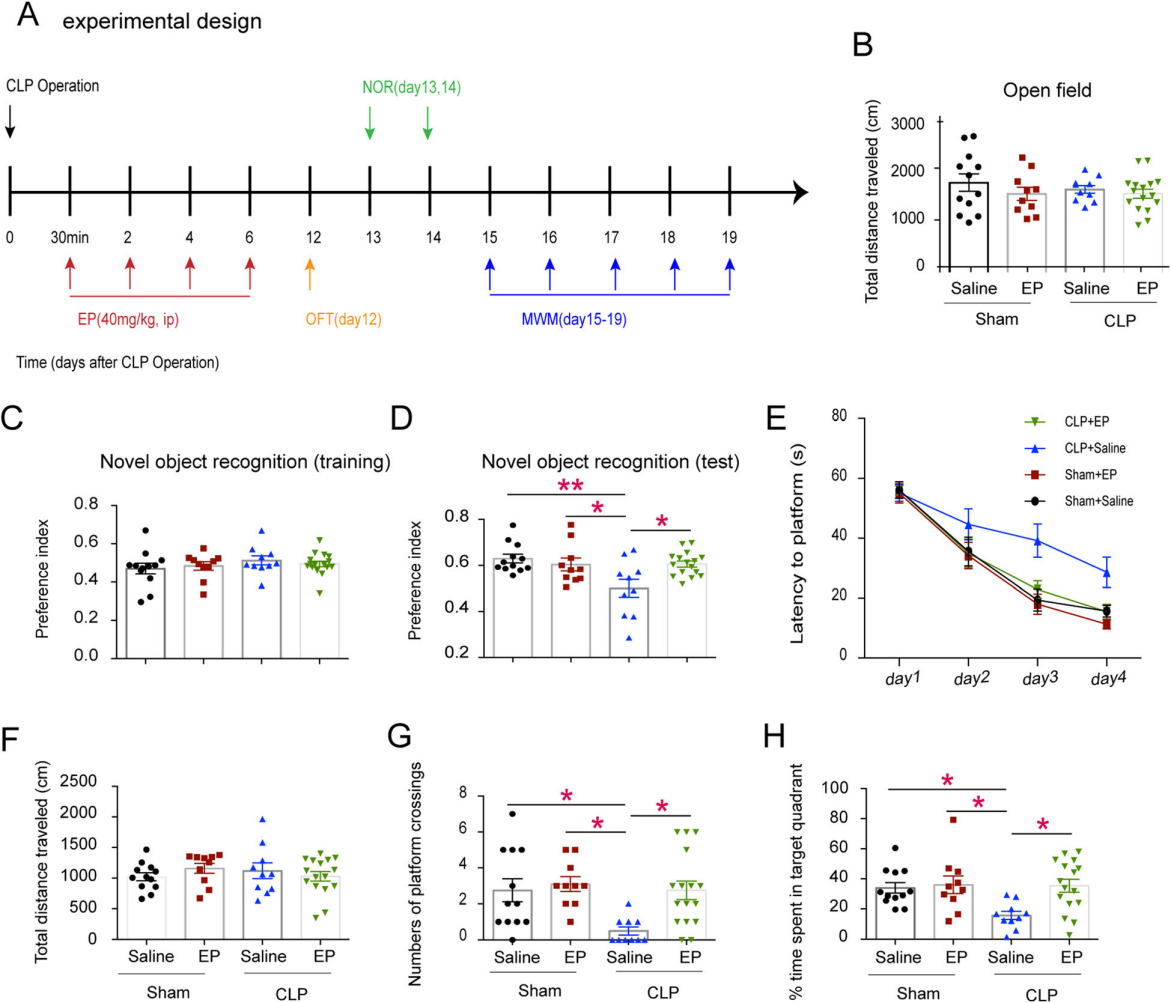
Ethyl pyruvate can improve cognitive dysfunction in septic mice. **a** Strategy of experiment. Eight-week-old male C57BL/6 J mice were subjected to CLP Operation, followed by the intervention of EP (40 mg/kg) or vehicle (PBS) once every 2 days during the infection period. **b** Traveled distance of the shamed or the septic mice treated with EP (40 mg/kg) or vehicle (PBS) in the open field test. **c** The preference indices of the four groups of mice in the training stage of novel object recognition test. **d** The preference indices of the four groups of mice in the testing stage of novel object recognition test **e** The escaped latency to platform of the four groups of mice during the training (the 15th to 18th days after surgery). **f**, **g** and **h** showing Total distance traveled, numbers of crossings through the platform area, and time percent spent in the target quadrant in probe trail, respectively. Data are presented as mean ± standard error of mean (SEM) (*n* ≥ 10 mice/ group) and compared by one-way ANOVA and post-hoc test. *p < 0.05; **p < 0.01

To determine whether ethyl pyruvate could improve learning and memory dysfunction in septic mice, we used NOR and MWM test after the mice subjected to moderate CLP or sham operation. After the mouse restored its mobility (Day 12, Fig. 1b), we carried out NOR test (Day 13) which was followed by MWM test (Day 15). We found that the mice in the CLP group had a discrimination index around 0.5 in the test phase (Fig. 1d), which indicated that the mice lost the memory of the old object and were unable to identify the novel object. The mice in the EP-treated and sham group had discrimination indices over 0.6, which significantly higher than that of the CLP group. In addition, the CLP rendered mice reduced capacity to find the underline platform during both training and test stages as compared to the sham group. EP treatment significantly restored the learning and memory after CLP (Fig. 1e-h). Thus, Ethyl pyruvate attenuates cognitive decline in experimental sepsis..

### Ethyl pyruvate attenuates microglia activation and restores formation of neonatal neurons in septic mice

**Fig. 2:**
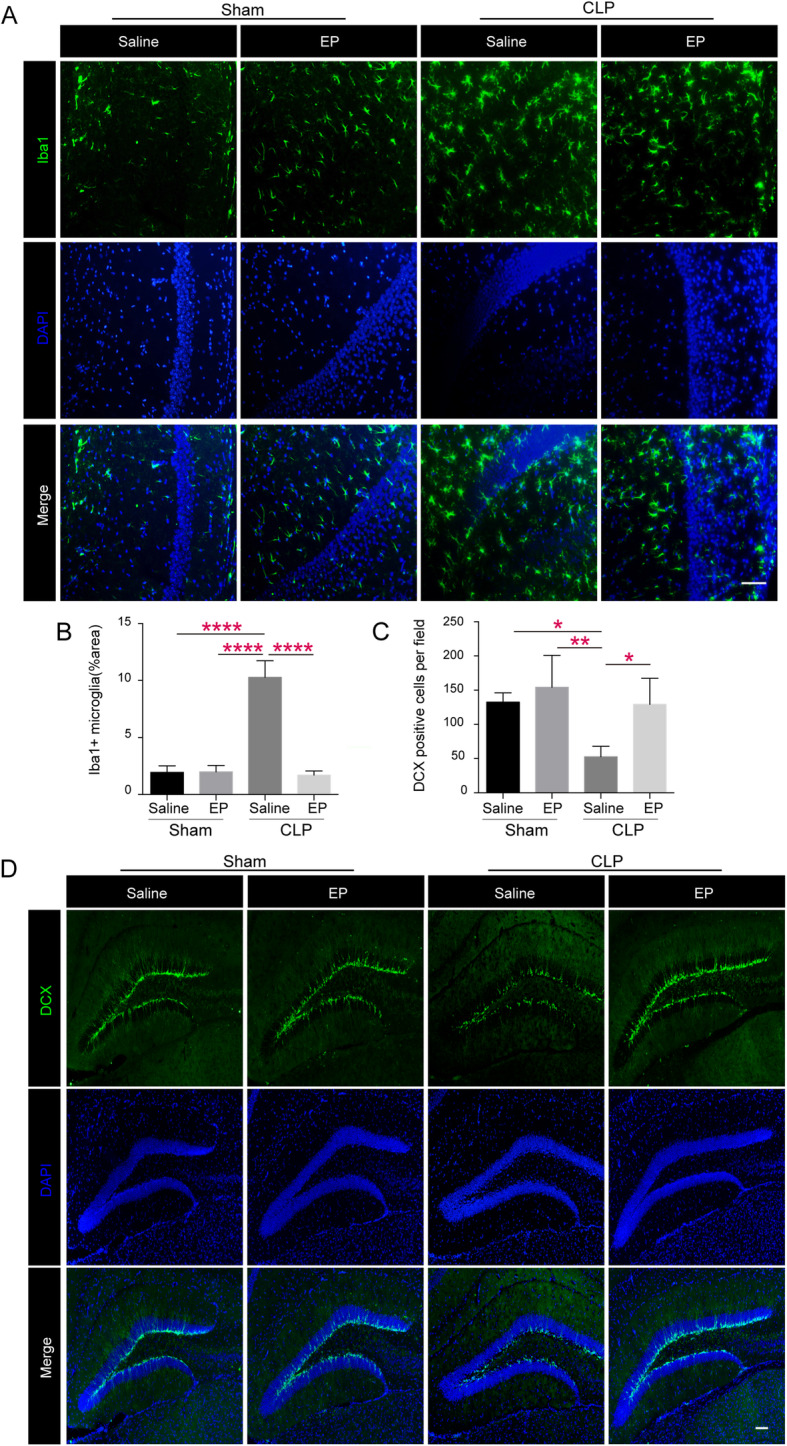
Ethyl pyruvate attenuates microglia activation and restores formation of neonatal neurons in septic mice. **a** Microglia activation of shamed or septic mice treated with EP (40 mg/kg) or vehicle (PBS) was assessed by Iba-1 immunofluorescence on postoperative day 12. Photomicrographs of CA1 areas of the hippocampus are shown infection activated microglia as noted by morphological changes on day12, including enlargement of cell bodies, unsmooth synapse, and so on, which was attenuated by EP treatment. Scale bar = 50 μm. **b** & **c** showing area percentage of activated microglia in the CA1 region and DCX positive cells in the dentate genus (DG) of septic mice hippocampus in Sham+Saline, Sham+EP, CLP + Saline and CLP + EP groups. Data presented as mean ± SEM (*n* ≥ 3 mice/ group) and compared by one-way ANOVA and post-hoc test, * *P* < 0.05, **P < 0.01, ****P < 0.0001. **d** Photomicrographs of representative DCX immunofluorescence (Doublecortin, green, neurogenesis) of shamed or septic mice treated with EP (40 mg/kg) or vehicle (PBS) in the DG areas on postoperative day 12. Scale bar =100 μm

Next we investigate the mechanisms by which EP attenuates cognitive dysfunction in sepsis, we first assessed the activation of microglia, which is one of the most important signs of neuro-inflammation. In young adult mice, the activation of microglial cells in the CA1 region was significantly increased after CLP, and the activation of microglial cells was significantly decreased after EP administration (Fig. 2a&b). It has been reported that decreased neurogenesis in the granular cell layer of the dentate gyrus is a causal factor of cognitive dysfunction in a series of encephalopathy (Abe 2000; Kim and Diamond 2002; Monje et al. 2002; Nixon and Crews 2010; Encinas et al. 2011). To test whether EP treatment exerts a protective role in improving neurogenesis in SAE, we detected the expression of Doublecortin (DCX), the marker of newborn neuron, in the dentate gyrus (DG) region using immunofluorescence. We found that the level of DCX in the DG region after CLP was significantly decreased, and that EP treatment restored the expression of DCX after CLP (Fig. 2c&d). These results demonstrated that EP might play a protective role in SAE by promoting neurogenesis and inhibiting the microglia activation in sepsis.

### Ethyl pyruvate inhibits NLRP3 inflammasome activation in mouse hippocampus and microglia

**Fig. 3:**
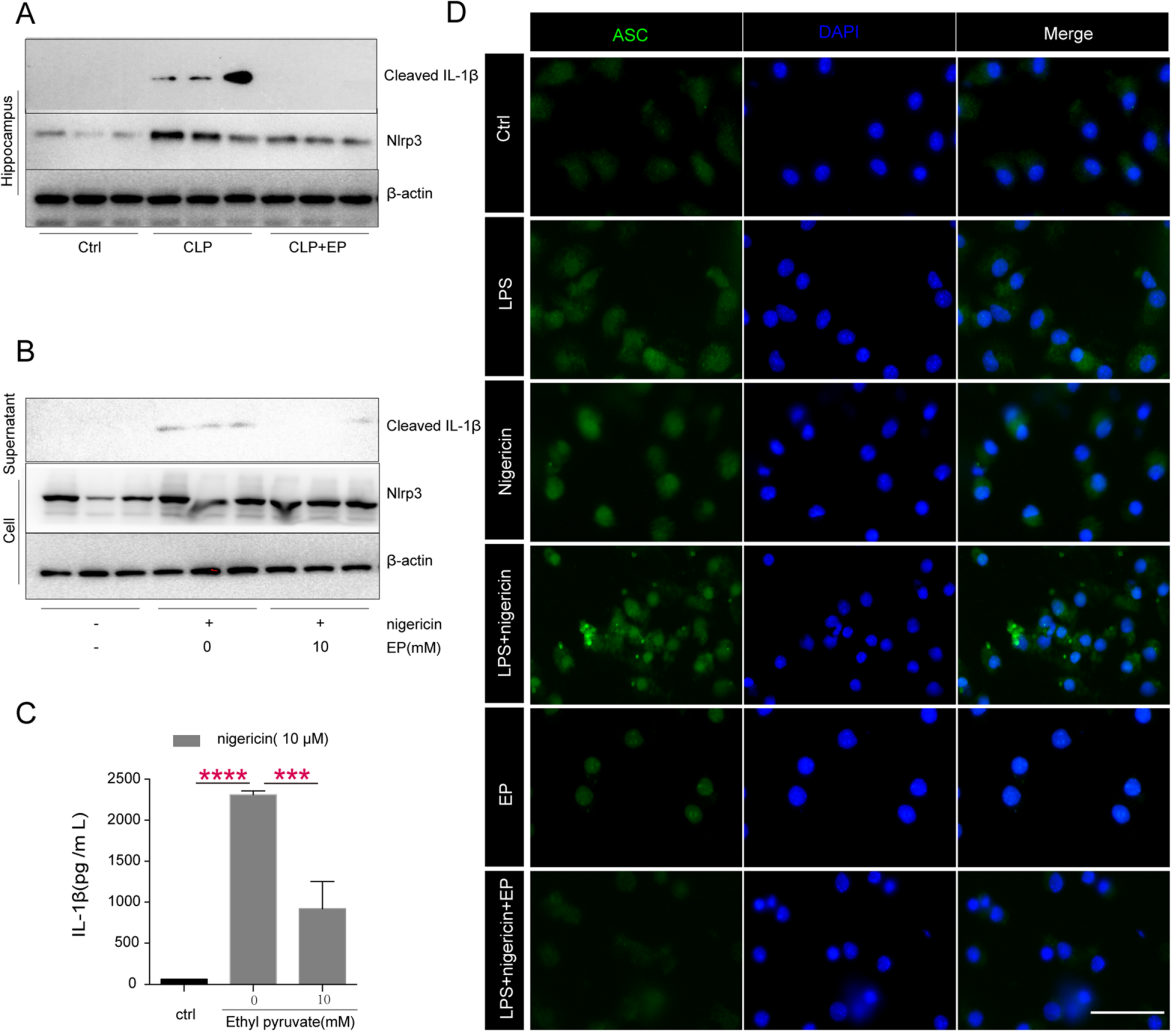
Ethyl pyruvate inhibits NLRP3 agonists-induced inflammasome activation in mouse microglia. **a** The expression of NLRP3 in hippocampus were assessed by Western-blot (**b**) mouse microglia were primed with ultra-pure LPS (100 ng/ml) for 3 h in the presence or the absence of EP (10 mM), and then stimulated with nigericin (10 μM) for 30 min. The release of IL-1β in supernatants and expression of NLRP3 in cell were assessed by Western-blot. **c** Levels of IL-1β in the culture medium were determined by ELISA. Data presented as mean ± SEM (*n* = 3 independent repeats/ group) and compared by one-way ANOVA and post-hoc test, ***P < 0.001, ****P < 0.0001. **d** immunofluorescence of ASC speck in LPS-primed microglia after incubation with nigericin (10 μM) for 15 min in the presence or the absence of ethyl pyruvate (10 mM). Shown in panel **c** are representative images of normal and ASC speck. Scale bars, 50 μm

To investigate the effect of EP to NLRP3 inflammsome in the SAE, the levels of NLRP3 of hippocampus in the mice challenged with or without CLP were determined via WB. The results suggested that the CLP model boosted the level of NLRP3 in the hippocampus, and EP significantly restored the increase of NLRP3 (Fig. 3a). In addition, our previous study suggests that EP inhibits the NLRP3 inflammasome activation in mouse macrophages (Li et al. 2018). To determine whether ethyl pyruvate (EP) also inhibits the NLRP3 inflammasome activation in mouse microglia, LPS-primed mouse microglia were stimulated with nigericin in the presence or the absence of EP. EP administration notably inhibited cleavage of pro-IL-1β at the concentration of 10 mM (Fig. 3b&c). Furthermore, EP significantly inhibits ASC speck formation in LPS-primed mouse microglia induced by nigericin (Fig. 3d). These results indicate that EP inhibits the NLRP3 inflammasome activation in mouse hippocampus and microglia.

### Ethyl pyruvate treatment attenuates SAE through inhibition of the activation of NLRP3 inflammasome

**Fig. 4:**
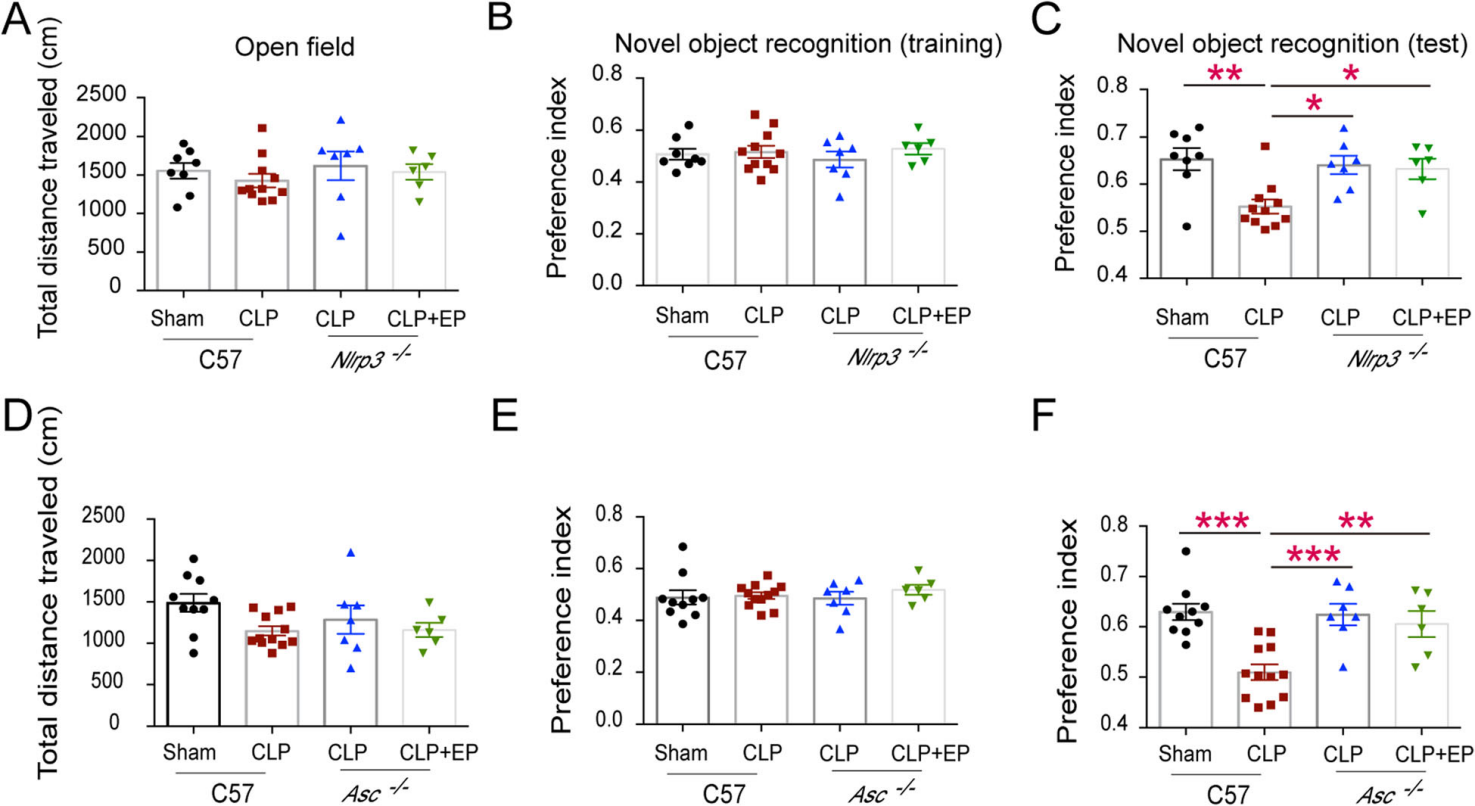
NLRP3 or ASC deficiency restored learning and memory impairment in septic mice. **a** Traveled distance of septic WT mice and septic *Nlrp3*^*−/−*^ mice in the open field test. **b** The preference indices of WT mice and *Nlrp3*^*−/−*^ mice subjected to CLP operation in the training stage of novel object recognition test. **c** The preference indices of the four groups of mice in the testing stage of novel object recognition test. **d** Traveled distance of septic WT mice and septic *Asc*^*−/−*^ mice in the open field test. **e** The preference indices of WT mice and *Asc*^*−/−*^ mice subjected to CLP operation in the training stage of novel object recognition test. **f** The preference indices of the four groups of mice in the testing stage of novel object recognition test. Data presented as mean ± SEM (*n* ≥ 6 mice/ group) and compared by one-way ANOVA and post-hoc test, * *P* < 0.05, **P < 0.01, ***P < 0.001

**Fig. 5:**
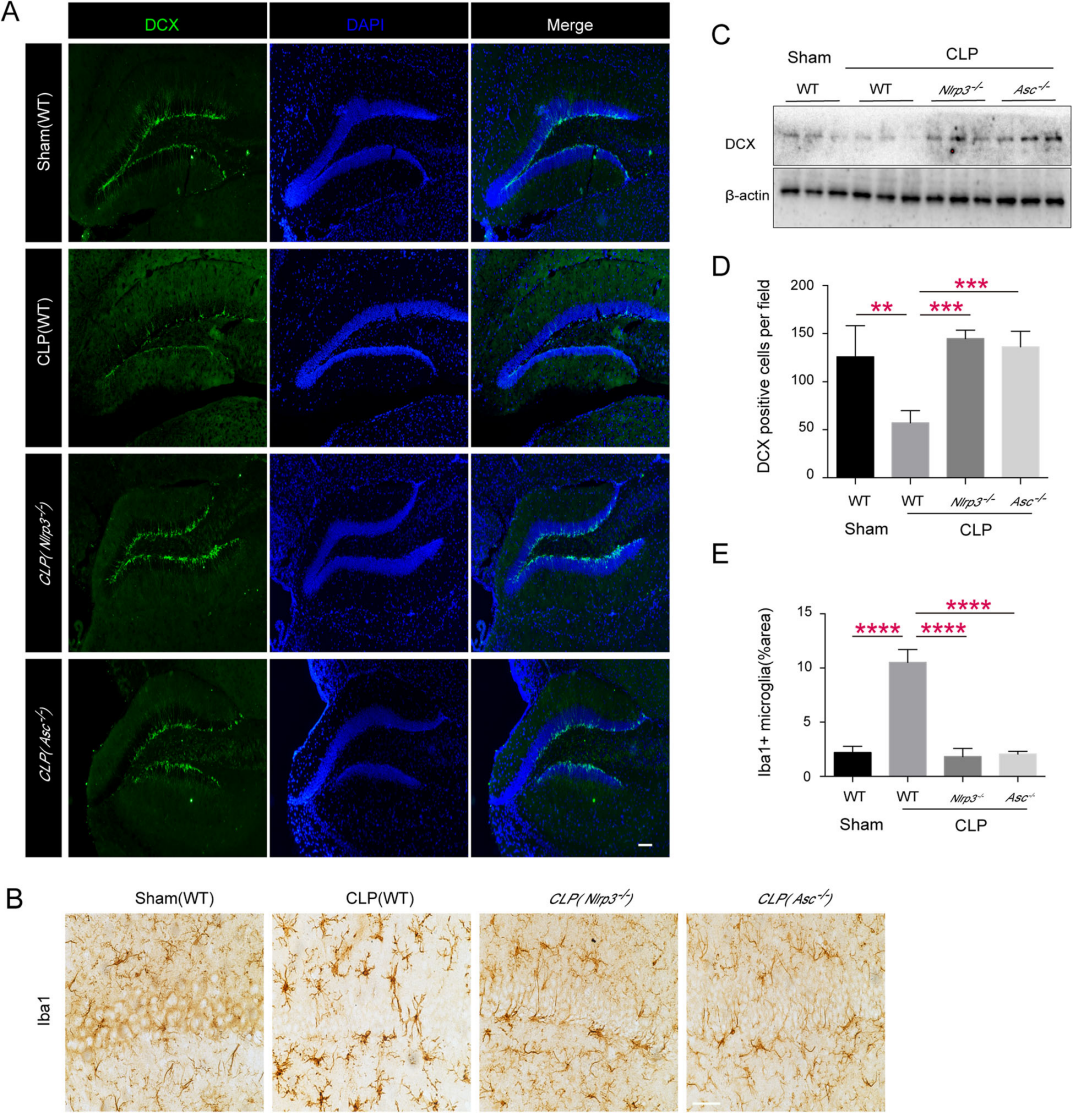
Ethyl pyruvate specifically inhibits NLRP3 inflammasome activation in septic mice. Representative DCX immunofluorescence in the DG areas (**a**) and Iba-1 immunohistochemical in the CA1 areas (**b**) of sham groups, WT groups, *Nlrp3*^*−/−*^ groups and *Asc*^*−/−*^ groups on postoperative day 12. Scale bar = 50 μm for Iba1, and Scale bar =100 μm for DCX. **c** The levels of DCX in the hippocampus of septic mice in sham groups, WT groups, *Nlrp3*^*−/−*^ groups and *Asc*^*−/−*^ groups were assessed by Western-blot on postoperative day 12. **d** & **e** showing area percentage of activated microglia in the CA1 region and DCX positive cells in dentate genus (DG) of septic mice hippocampus in sham groups, WT groups, *Nlrp3*^*−/−*^ groups and *Asc*^*−/−*^ groups. Data presented as mean ± SEM (*n* ≥ 3 mice/ group) and compared by one-way ANOVA and post-hoc test, **P < 0.01, ***P < 0.001, ****P < 0.0001

Previous studies showed that ethyl pyruvate inhibited the activation of NLRP3 inflammasomes at the cell level. Thus, we speculate that ethyl pyruvate might inhibit the activation of the NLRP3 inflammasomes in the brain of septic mice. In order to further prove that EP does play a protective role through inhibiting NLRP3 inflammasome, we carried out further experiments on *Nlrp3* and *Asc* knockout mice. We found that NLRP3/ASC deficient mice showed the same degree of cognitive impairment as WT intact mice, and there was no additive protective effect after EP administration (Fig. 4). In addition, we found that *Nlrp3* and *Asc* knockout mice displayed better neurogenesis than WT septic mice (Fig. 5a&c&d), and the activation of microglia was significantly decreased (Fig. 5b&e). These results suggest that EP may inhibit the activation of microglial cells and ameliorate cognitive dysfunction in septic mice by inhibiting the activation of NLRP3 inflammasome.

### Intrathecal injection of EP rescued the SAE in the CLP model

**Fig. 6:**
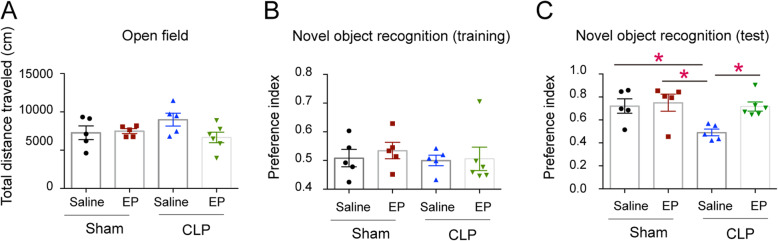
Intrathecal injection of EP rescued the SAE in the CLP model. **a** Traveled distance of shamed or septic mice treated with EP (2.5 mg/kg) or vehicle (PBS) in the open field test. **b** The preference indices of the four groups of mice in the training stage of novel object recognition test. **c** The preference indices of the four groups of mice in the testing stage of novel object recognition test. Data presented as mean ± SEM (*n* ≥ 5 mice/ group) and compared by one-way ANOVA and post-hoc test, * *P* < 0.05

To test whether EP improves SAE by directly affecting central nervous system or by improving systemic sepsis (secondary effect), we rescued the CLP-challenged mice with the intrathecal injection of EP. NOR tests were conducted to assess the cognitive function in mice after the mice subjected to moderate CLP or sham operation. We found that EP local administration rescued the SAE in the CLP model. The results suggested that local administration of EP significantly restored cognitive impairment in the CLP model (Fig. 6), which indicates that EP, at least in part, has direct effect on brain to improve SAE.

## Discussion

In this study, we found that Ethyl pyruvate treatment significantly attenuated cognitive decline, microglia activation, and impaired neurogenesis in experimental sepsis, at least in part, through inhibition of the NLRP3 inflammasome..

Aberrant activation of the NLRP3 inflammasome in the central nerve system leads to abnormal function of microglia, which contributes to a number of neurodegenerative disorders (Polazzi and Contestabile 2002; Block et al. 2007). The activated microglia can release a range of pro-inflammatory factors, particularly TNF-α and IL-1β (Delgado et al. 1998; Delgado and Ganea 2003; Qin et al. 2004; Burguillos et al. 2011). Previous studies have documented that the activated microglia disrupts neurogenesis, which is implicated in cognitive impairment and mood disorders (Kohman and Rhodes). In the present study, EP significantly restores the cognitive function in sepsis model by inhibiting microglia activation.

The inflammatory cytokines, in particular IL-1β, should be the major culprit in the decline of neurogenesis (Kohman and Rhodes). It has been reported that neural progenitor cells (NPCs) express IL-1 receptor and high levels of IL-1β significantly inhibits the proliferation of NPCs and the growth of neurospheres (Green et al. 2012). In addition, the presence of IL-1β facilitates the differentiation of NPCs into astrocyte rather than neurons, which eventually results in the reduction of neurogenesis (Green et al. 2012). These in-vitro studies are phenocopied by in-vivo studies in which over-expression of IL-1β decreases the number of DCX+ cells (newborn neurons) in the granular layer of the hippocampus (Wu et al. 2012). Importantly, it is reported that neuroinflammation contributes to the pathogenesis of long-term cognitive impairment of SAE (Wu et al. 2013; Da-ming et al. 2016; Fu et al. 2018). The NLRP3 inflammasome is an intracellular supramolecular complex composed of NLRP3, ASC and caspase-1. Upon activation, ASC is self-assembled into ASC speck (Fernandes-Alnemri et al. 2007), and then activate pro-caspase-1 by proximal induced autocatalytic activation (Hoss et al. 2016). Finally, activated caspase-1 triggers the maturation of interleukin-1 (IL-1) family cytokines (e.g. IL-1β and IL-18) and facilitates the release of these cytokines via the N-terminal of GSDMD constituted transmembrane pores. Thus, the activation of NLRP3 inflammasome can result in a hyperactive status of immune cells and facilitate the release of IL-1β(Kohman and Rhodes 2013), and may facilitate the disruption of neurogenesis. Previous works reveal that the NLRP3 inflammasome contributes to the pathogenesis of a number of neurodegenerative diseases including Alzheimer’s (Heneka et al. 2014; Walsh et al. 2014; Zhang and Jiang 2015), Parkinson’s disease (Franchi et al. 2010; Amor et al. 2014; Yan et al. 2015) and Multiple Sclerosis (MS) (Jha et al. 2010; Guo et al. 2015; Weinberg et al. 2015; Yeung et al. 2015; Matthews et al. 2016). In addition, the excessive activation of NLRP3 inflammasome promotes memory loss in these diseases (Miller et al. 2009; Kreisel et al. 2014; Zhang et al. 2014; Sui et al. 2016). In line with these observations, we found that ethyl pyruvate treatment significantly inhibited NLRP3 inflammasome activation and IL-1β release of microglia, and improved the decline of DCX positive cells and the cognition impairment in the CLP model. Thus, EP attenuates SAE through inhibition of the activation of NLRP3 inflammasome.

Ethyl pyruvate (EP), an anti-inflammatory reagent (O'Donnell-Tormey et al. 1987; Han et al. 2005), is considered as a potential therapeutic drug for various diseases or disorders, including burn injury (Huang et al. 2008), shock (Tawadrous et al. 2002), spinal cord injury (Genovese et al. 2009; Wang et al. 2009), hemorrhagic shock (Slovin et al. 2001), acute endotoxemia (Wang et al. 1999; Ulloa et al. 2002; Venkataraman et al. 2002), severe acute alcoholic hepatitis (Yang et al. 2003) and multiple models of ischemia-reperfusion (Tawadrous et al. 2002; Woo et al. 2004). In sepsis model, EP improves the survival rate of mice when mice administered 24 h after the onset of sepsis (Ulloa et al. 2002). Other studies suggested that EP can attenuate multiorgan dysfunctions in endotoxemia or sepsis (Miyaji et al. 2003; Hauser et al. 2005). It is important to note that the safety of EP has been confirmed by the long-term application as a food additive (Fink 2003; Organization, F. A., and W. H. Organization 2010). Thus, EP might be a promising medicine in the protection against cognitive dysfunction in sepsis.

## Conclusions

In summary, our results suggest that ethyl pyruvate treatment might improve cognitive function in sepsis through inhibition of the NLRP3 inflammasome.

## Data Availability

All data generated or analyzed during this study are included in this published article.

## Abbreviations

SAE: Sepsis-associated encephalopathy
NLRP3: Pyrin domain-containing 3
IL: Interleukin
CLP: Cecal ligation and puncture
ASC: Apoptosis-associated speck-like protein containing a CARD
EP: Ethyl pyruvate
KO: Knockout
WT: Wild-type
IBA1: Ionized calcium binding adaptor molecule 1
DCX: Doublecortin
DG: Dentate gyrus
